# Comparing the diagnostic accuracy of cerebrospinal fluid biomarkers for Alzheimer’s disease: A novel meta‐analysis model

**DOI:** 10.1002/alz.085358

**Published:** 2025-01-09

**Authors:** Athena L Sheppard, Terry J Quinn, Sylwia Bujkiewicz, Rhiannon K Owen

**Affiliations:** ^1^ Swansea University, Swansea United Kingdom; ^2^ University of Glasgow, Glasgow United Kingdom; ^3^ University of Leicester, Leicester United Kingdom

## Abstract

**Background:**

The emergence of new biomarkers for Alzheimer’s disease is accompanied by the urgent need to evaluate their diagnostic performance in comparison to existing technologies. Meta‐analysis methods make best use of the available evidence by synthesizing all relevant published studies. Meta‐analytic models that estimate comparative diagnostic accuracy are key to informing healthcare decision‐making around which tests should be used in clinical practice. To produce robust and precise estimates of sensitivity and specificity, models must take into account associations between multiple tests evaluated in the same patient group.

**Method:**

A novel Bayesian meta‐analysis model for jointly synthesizing accuracy data on two diagnostic tests was developed using a motivating example in Alzheimer’s disease dementia. A statistical technique known as a copula was used to flexibly capture the associations between the two tests. Comparative diagnostic accuracy data was sourced through electronic searching of The Cochrane Library. The model was fit to studies comparing the diagnostic accuracy of cerebrospinal fluid (CSF) amyloid‐β 42 (Aβ42) and total tau (t‐tau) to detect patients with mild cognitive impairment who would convert to Alzheimer’s disease dementia.

**Result:**

CSF Aβ42 (80.6%; 95% credible interval [CrI] 70.8%, 88.3%) was more sensitive to detect Alzheimer’s disease dementia than CSF t‐tau (74.6%; 95% CrI 65.4%, 82.2%). However, CSF t‐tau demonstrated slightly higher specificity (75.5%; 95% CrI 64.9%, 84.4%) than CSF Aβ42 72.6% (95% CrI: 62.9%, 81.2%). In comparison to the meta‐regression approach currently recommended by evidence synthesis guidelines, which ignores associations between tests, the newly proposed model led to increased precision around summary sensitivity and specificity estimates.

**Conclusion:**

The proposed model reduces uncertainty around summary test accuracy, aiding decision‐making around diagnostic strategies for Alzheimer’s disease dementia. This novel methodological development is applicable to a range of diagnostic tests for Alzheimer’s disease, including newly emerging blood‐based biomarkers. Continued development of early and minimally‐invasive diagnostic biomarkers will require comprehensive evaluation in the context of existing tests, using appropriate statistical models that capture associations between tests.

